# Karyotype and genome size in *Euterpe* Mart. (Arecaceae) species

**DOI:** 10.3897/CompCytogen.v10i1.5522

**Published:** 2016-01-22

**Authors:** Ludmila Cristina Oliveira, Maria do Socorro Padilha de Oliveira, Lisete Chamma Davide, Giovana Augusta Torres

**Affiliations:** 1 Universidade Federal de Lavras, Campus Universitário, Caixa Postal 3037, CEP 37200-000, Lavras-MG, Brasil; 2 Embrapa Amazônia Oriental, Trav. Dr. Enéas Pinheiro, s/n°, Bairro Marco, CEP 66095-100, Caixa Postal 48, Belém-PA, Brasil

**Keywords:** C-value, interphase nucleus, chromosomal evolution, flow cytometry, Açaí palm

## Abstract

*Euterpe* (Martius, 1823), a genus from Central and South America, has species with high economic importance in Brazil, because of their palm heart and fruits, known as açaí berries. Breeding programs have been conducted to increase yield and establish cultivation systems to replace the extraction of wild material. These programs need basic information about the genome of these species to better explore the available genetic variability. The aim of this study was to compare *Euterpe edulis* (Martius, 1824), *Euterpe oleracea* (Martius, 1824) and *Euterpe precatoria* (Martius, 1842), with regard to karyotype, type of interphase nucleus and nuclear DNA amount. Metaphase chromosomes and interphase nuclei from root tip meristematic cells were obtained by the squashing technique and solid stained for microscope analysis. The DNA amount was estimated by flow cytometry. There were previous reports on the chromosome number of *Euterpe edulis* and *Euterpe oleracea*, but chromosome morphology of these two species and the whole karyotype of *Euterpe precatoria* are reported for the first time. The species have 2n=36, a number considered as a pleisomorphic feature in Arecoideae since the modern species, according to floral morphology, have the lowest chromosome number (2n=28 and 2n=30). The three *Euterpe* species also have the same type of interphase nuclei, classified as semi-reticulate. The species differed on karyotypic formulas, on localization of secondary constriction and genome size. The data suggest that the main forces driving *Euterpe* karyotype evolution were structural rearrangements, such as inversions and translocations that alter chromosome morphology, and either deletion or amplification that led to changes in chromosome size.

## Introduction

*Euterpe* (Martius, 1823) (Arecaceae-Arecoideae), is composed of seven species distributed from Central to South America (Henderson 1995). In Brazil, *Euterpe edulis* (Martius, 1824), *Euterpe oleracea* (Martius, 1824) and *Euterpe precatoria* (Martius, 1842) are considered the most important species of the genus due to their wide distribution and economic importance of their fruits and palm hearts, obtained mainly by extractive activity in Brazil (Castro 1992). The high commercial value of their products, especially of the açaí palm (*Euterpe oleracea*), has encouraged the development of genetic improvement programs to produce cultivars with higher yield and better quality of fruits and palm heart. In addition to the economic value, cultivation instead of extraction of wild material should favor the conservation of those species, which is urgent in the case of *Euterpe edulis* since it is a threatened species.

Cytogenetic data are critical for germplasm manipulation for such programs, especially when the use of interspecific hybrids is considered as a strategy to increase the variability and to incorporate alleles of interest (Bovi 1987). However, only the chromosome number of *Euterpe oleracea* and *Euterpe edulis* (2n=36) was reported in Môro et al. (1999), and there is no information on chromosome morphology. There are also no data regarding the interphase nucleus for the genus *Euterpe*. Röser (1994) studied 56 taxa belonging to six subfamilies of Arecaceae and found highly differentiated interphase nuclei, ranging from reticulate and semi-reticulated to an intermediate stage between semi-reticulate and areticulate.

Determination of genome size in plants has been recognized as a significant parameter for genomic characterization and may assist in evolutionary studies (Knight and Beaulieu 2008), genetic improvement (Doležel 1997), systematics and molecular and cellular biology (Bennet and Leitch 1995). Röser et al. (1997) used Feulgen densitometry to assess nuclear DNA amount in 83 species of palm trees, belonging to 53 genera. They observed a C-value range between 0.97 and 13.91 pg, a variation of approximately 14.3 times in genome size. *Euterpe precatoria*, was the single species analyzed, showing 5.31 pg (1C).

Therefore, the aims of this study were to compare karyotype, interphase nucleus pattern and genome size of *Euterpe edulis*, *Euterpe oleracea* and *Euterpe precatoria* and discuss the karyotypic evolution within the genus.

## Material and methods

### Genetic material

The Açaí Palm Germplasm Bank (Banco de Germoplasma de Açaizeiro - BAG-Açaí), from Embrapa Amazônia Oriental in Belém-PA, Brazil, provided seeds from five specimens of *Euterpe oleracea* and *Euterpe precatoria*. The company Infrater Engenharia LTDA, headquartered in Ipatinga-MG, donated seeds from five specimens of *Euterpe edulis*.

### Karyotype analysis

Roots originating from germinated seeds were pre-treated with 2 mM 8-hydroxyquinoline for 7 h at 4 °C. Slides were prepared by the squashing technique following cell wall digestion with pectinase/cellulase (100/200U) solution at 37 °C for 1.5 h. Staining was performed with 1% propionic carmine for the analysis of the mitotic metaphases and 5% Giemsa for the evaluation of the interphase nuclei. The images were acquired in a bright-field microscope (Leica DMLS) equipped with a digital camera (Nikon Digital Sight DS-Fi1).

The short and long arms (SA and LA, respectively) of chromosomes were measured using the IMAGE TOOL 3.00 program from UTHSCA (University of Texas Health Science Center in San Antonio). The mean lengths of SA and LA of each chromosome were obtained from measurement of five different metaphases from each species and were used to prepare the ideograms. The chromosome total length (TL = SA + LA), the haploid complement total length (HCTL = ∑Lti), the centromeric index (CI=[SA/(SA+LA)]×100) were calculated. The chromosomes were classified based on their centromere position according to Guerra (1986). The karyotype asymmetry was calculated according to Romero Zarco (1986).

### Flow cytometry

The nuclear DNA amount was estimated by flow cytometry using leaf tissue from three specimens per species. Each sample contained 20-30 mg of young leaves of the target species mixed with young leaves of *Vicia faba* L. cv. Inovec the internal reference standard with 1C=13.33 pg (Johnston et al. 1999). The samples were ground on a Petri dish with 1 mL of ice-cold Marie buffer (Doležel 1997). The final nuclear suspension was mixed with 25 µL of propidium iodide (1 mg/mL). At least 10.000 nuclei per sample were analyzed in a FacsCalibur cytometer (Becton Dickinson). Histograms were acquired using CELL QUEST PROGRAM (Becton, Dickinson and Company, San Jose, CA, USA) and analyzed using the WINMDI 2.8 software (2009).

### Statistical analysis

The HCTL and nuclear DNA amount data were submitted to analysis of variance and the means compared by the Tukey’s test at 5% probability using the SISVAR statistical program.

## Results

**Figure 1.**
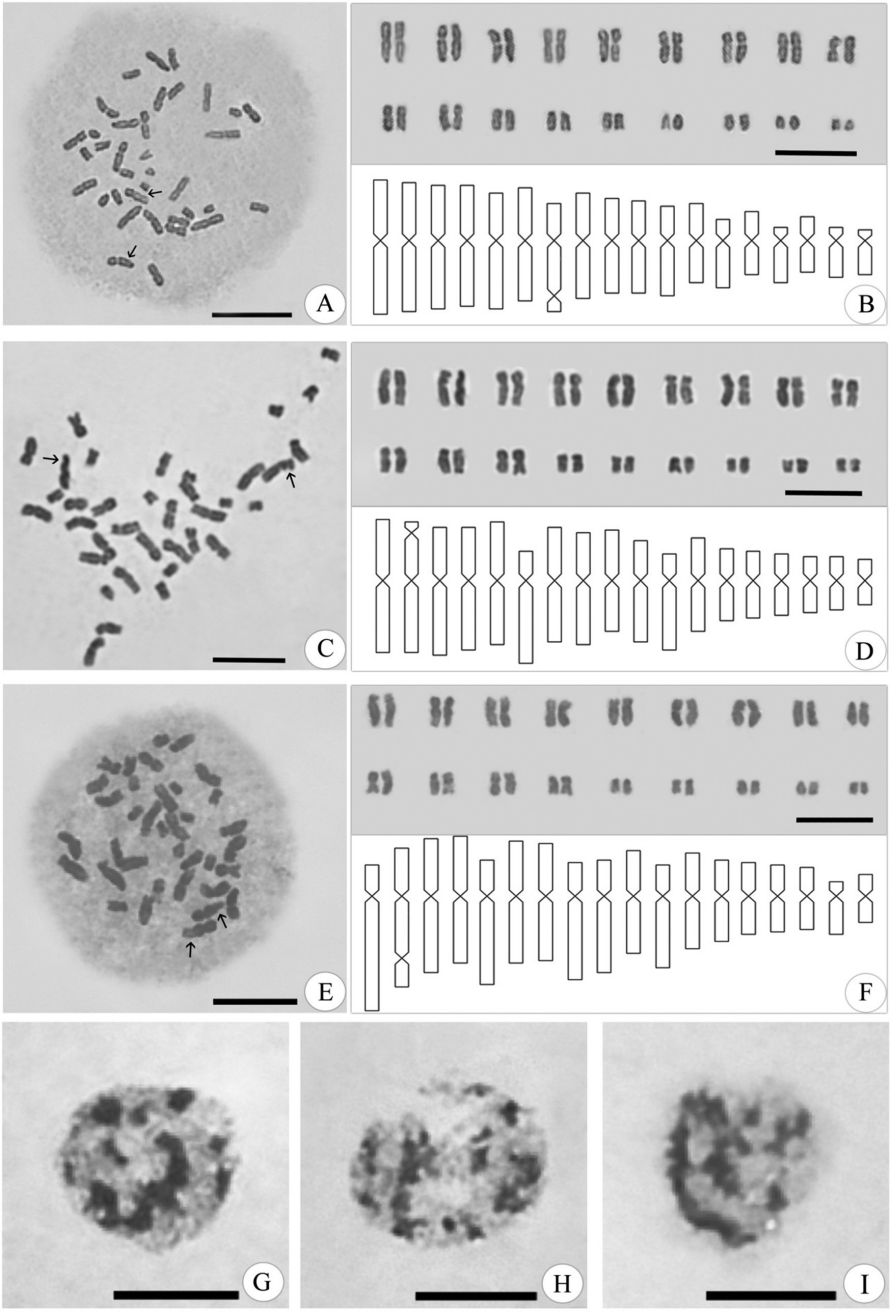
Mitotic metaphases, karyograms and idiogram of *Euterpe* species with 2n=36 chromosomes. *Euterpe edulis* (**A–B**), *Euterpe oleracea* (**C-D**) and *Euterpe precatoria* (**E–F**). Arrows indicate secondary constrictions. Semi-reticulate interphase nuclei of *Euterpe edulis* (**G**), *Euterpe oleracea* (**H**) and *Euterpe precatoria* (**I**). Bar: 10 µm.

*Euterpe edulis*, *Euterpe oleracea* and *Euterpe precatoria* have the same chromosome number (2n=36), similar chromosome sizes and differ regarding chromosome morphology (Fig. 1a–f). Chromosome total length decreases gradually (Fig. 1b, d, f), ranging from 4.1 to 1.29 in *Euterpe edulis*; 4.08 to 1.39 in *Euterpe oleracea* and 4.7 to 1.5 in *Euterpe precatoria*. Variation in chromosome size within the karyotype is very similar among the species as pointed by A2 index (Table 1). The species differ mainly in chromosome morphology and genome size. As indicated by the karyotype formula and A1 index (Table 1), *Euterpe oleracea* karyotype is the most divergent one, being more symmetric than the two others.

**Table 1.** C-value, haploid complement total length (HCTL), karyotype formula and asymmetry indexes (Romero Zarco 1986) of *Euterpe* species.

Species	C-value (pg)	HCTL (µm)	Karyotype formula	A1	A2
***Euterpe edulis***	4.09 a	49.60a	12M + 3SM + 3A	0.327	0.329
***Euterpe oleracea***	4.22 a	51.30a	14M + 4SM	0.259	0.327
***Euterpe precatoria***	4.71 b	59.39b	11M + 6SM + 1A	0.346	0.315

Means followed by the same letter do not differ statistically by the Tukey’s test at 5% probability.

The chromosome pairs from 1 to 12 of *Euterpe edulis* and *Euterpe oleracea* are quite similar morphologically, and eight have the same classification, seven metacentric and one submetacentric. The same pairs are quite different in *Euterpe precatoria*, which has the highest number of submetacentric chromosomes and one acrocentric pair, the largest and only pair of chromosomes with that morphology (Fig. 1b,d,f).

The chromosome pairs from 13 to 18, except 17, are all metacentric in *Euterpe oleracea* and *Euterpe precatoria*. The same pairs are different in *Euterpe edulis*, with two pairs of acrocentric chromosomes (15 and 18) and one submetacentric chromosome (13). Chromosome pair 17 is the only one in the complements that differs regarding centromere position in all three species; it is metacentric in *Euterpe oleracea*, submetacentric in *Euterpe precatoria* and acrocentric in *Euterpe edulis* (Fig. 1b, d, f).

One pair of chromosomes bears one secondary constriction in all three species. It is located on the long arm of pair seven (submetacentric) in *Euterpe edulis*, in the short arm of pair two (metacentric) in *Euterpe oleracea* and on the long arm of pair two (submetacentric) in *Euterpe precatoria* (Fig. 1b, d, f).

*Euterpe precatoria* showed HCTL and DNA content significantly higher than that of *Euterpe edulis* and *Euterpe oleracea* (Table 1). The mean coefficient of variation (CV) of flow cytometry data was 0.52%, which demonstrates the reliability of DNA amount estimation, since only CVs up to 2% indicate high quality analysis (Marie and Brow 1993). The genome size is estimated in 4Gb, 4.13Gb and 4.61Gb for *Euterpe edulis*, *Euterpe oleracea* and *Euterpe precatoria*, using the coversion rate of 1pg = 978Mb.

Interphase nuclei were quite similar, classified as semi-reticulate due the formation of strongly pigmented chromatin structures with irregular contours (Fig. 1g, h, i).

## Discussion

The chromosome number of *Euterpe edulis* and *Euterpe oleracea*, 2n=36, was also reported by Môro et al. (1999), while for *Euterpe precatoria*, also 2n=36, this is the first report. Considering that *Euterpe microcarpa* also has 2n=36 (Röser 1994), *Euterpe* shows high stability in chromosome number. In Arecoideae, 2n=36 is the highest number found, but also the most rare, being characteristic of New world species. It is considered a pleisomorphic karyological feature, since the modern species, considering floral morphology, have the lowest chromosome number (2n=30 and 2n=28). The hypothesis is that starting from 2n=36 (basic number x=18) different and independent reduced dysploid series diverged not only in Arecoideae (2n=28 to 2n=36), but also in Coryphoideae (2n=28 to 2n=36) and Calamoideae (2n=26 to 2n=36) (Röser 1994).

The analyzed karyotypes showed differences in centromere and secondary constriction position. The chromosomes may differ in terms of centromere position, according to Stebbins (1971), through pericentric inversions or uneven translocations, rearrangements that substantially contribute to the increase of karyotype asymmetry. Our results for karyotype asymmetry measure (Tab 1) revealed that the karyotypes are quite similar for chromosome size and differ for chromosome morphology. Along with stability in chromosome number, 2n=36 for all *Euterpe* species studied, these data indicate that the rearrangements may be responsible for karyotype variation among the three *Euterpe* species studied.

Most karyotype studies on palm trees do not include data on the number and location of secondary constrictions. The study performed by Röser (1993) describes the karyotypes of 13 species belonging to 13 different genera of the Coryphoideae subfamily, describing the presence of secondary constrictions in 10 of them. The author found a single pair of chromosomes bearing secondary constriction in eight species: *Livistona chinensis* Brown, 1810, *Pritchardia thurstonii* Mueller & Drude, 1887, *Brahea edulis* Wendland ex Watson, 1876, *Copernicia macroglossa* Wendland, 1907, *Washingtonia robusta* Wendland, 1883, *Sabal minor* (Jacquin, 1805), *Bismarckia nobilis* Hildebrandt & Wendland, 1881 and *Phoenix canariensis* Chabaud, 1882. The other studied species showed two pairs or no pairs of chromosomes with secondary constriction.

The evolutionary direction of karyological changes was shown to be from reticulate to areticulate interphase nuclei when comparing the systematic classification of some Arecaceae subfamilies, ​​mainly based on plant morphological characteristics, with the characterization based on the interphase nuclei and karyotypes (Röser 1994). Therefore, it is possible to infer that, regarding the organization of the interphase nucleus, the three *Euterpe* species have an intermediate level of evolution within the family.

Nuclear DNA quantification, when combined with interphase nucleus characterization and karyological data, may enable differentiation because it allows for the detection of small differences in the DNA amount between species. Those differences make it possible to infer chromosome rearrangements that may be too small to affect the physical structure of the chromosomes. Furthermore, according to Schifino-Wittmann (2001), data on the nuclear DNA amounts of species assist in the management of large germplasm collections and the control of ploidy levels in progenies generated by crosses.

Röser, Johnson and Hanson (1997) reported, through Feulgen densitometry, 5.31 pg of nuclear DNA (1C) in *Euterpe precatoria*, 0.6 pg higher than the value reported in this study. According to Schifino-Wittmann (2001), both the nuclear genome plasticity and certain aspects of the methodologies applied must be considered when the DNA amounts assessed by different authors are divergent. Regarding the methodology, the flow cytometry estimates using propidium iodide (PI) has shown to be highly correlated with Feulgen densitometry ones. However, despite of being a well stablished method for DNA quantification, Feulgen densitometry has some critical points in the procedure that can affect its precision (Doležel et al. 1998), which can explain the difference between our estimate and the one in the literature.

The comparison among the three species with respect to the nuclear DNA amount and total length of the haploid complement showed that *Euterpe precatoria* has a larger genome than *Euterpe edulis* and *Euterpe oleracea*. Considering that they showed similar inner variation in chromosome size, the difference in DNA amount can be better explained by increase or decrease of size, by amplification or deletion, respectively, involving most of chromosomes.

Differences in genome size and chromosome morphology among the three *Euterpe* species revealed that structural rearrangements were the main force driving karyotype evolution in the genus. Higher resolution techniques, like chromosome banding and molecular hybridization (FISH) should be used to unravel the mechanisms involved.
